# The Study of 3D Printing-Assisted Electrospinning Technology in Producing Tissue Regeneration Polymer-Fibroin Scaffold for Ureter Repair

**DOI:** 10.5152/tud.2022.21217

**Published:** 2022-03-01

**Authors:** Han-Yen Hu, Chia-Lun Wu, Cheng-Shuo Huang, Meng-Yi Bai, Dah-Shyong Yu

**Affiliations:** 1 National Taiwan University of Science and Technology, Graduate Institute of Biomedical Engineering, Taipei, Taiwan, ROC; 2 Graduate Institute of Life Sciences, National Defense Medical Center, Taipei, Taiwan, ROC; 3 Graduate Institute of Pathology and Parasitology, National Defense Medical Center, Taipei, Taiwan, ROC; 4 Uro-Oncology Laboratory, Division of Urology, Department of Surgery, Tri-Service General Hospital, National Defense Medical Center, Taipei, Taiwan, ROC Equally contributed.

**Keywords:** 3D printing; electrospinning; polycaprolactone; fibroin; scaffold; tissue regeneration; ureteral injury.

## Abstract

**Objective:**

Long segment ureteral lesion with obstruction is a clinically difficult issue for recovering and maintaining organ or tissue function. Regeneration medicine using various biomaterials as a scaffold in supporting tissue regrowth is emerging. We developed this customized scaffold using electrospinning and 3-dimensional assistance and expected that it may provide an alternative biomaterial for ureter defect repair.

**Material and Methods:**

Our study synthesized polycaprolactone and silk fibroin combination as biomaterial scaffolds. The differences in physicochemical properties and biocompatibility of polycaprolactone–silk fibroin bio-scaffolds prepared by electrospinning alone and 3-dimensional printing combined with electrospinning in proper ratios were compared and characterized. SV-HUC-1 uroepithelial cells cultured in polycaprolactone–silk fibroin (4 : 6) scaffolds were observed under a scanning electron microscope and using calcein-acetomethoxy and propidium iodide stain. The ex vivo resected healthy human ureteral segment tissue was anastomosed with the polycaprolactone–silk fibroin scaffolds and cultured in an ex vivo bath for 2 weeks. The cellular growth on the polycaprolactone–silk fibroin scaffold was observed microscopically. In the New Zealand white rabbit model, we performed a 1/5 ratio (2 cm out of 10 cm) defect replacement of the unilateral ureter. After 7 weeks, the rabbits were sacrificed and the implanted ureter scaffolds were resected for tissue sectioning and the cellular growth was observed by hematoxylin and eosin and Masson staining.

**Results:**

When the proportion of silk fibroin was increased and the 3-dimensional electrospinning method was used, both the size and diameter of nanofiber holes were increased in the polycaprolactone–silk fibroin scaffold. Scanning electron microscope and fluorescent stain revealed that cultured 3T3 and SV-HUC-1 uroepithelial cells could electively penetrate inside the polycaprolactone–silk fibroin (4 : 6) nanoﬁbrous scaffolds in 3 days. The polycaprolactone–silk fibroin scaffold anastomosis in an ex vivo bath showed cellular growth stably along the scaffold for 2 weeks, and most of the cells grow along with the outboard of the scaffold in layers. In an animal model, different layered cells can be observed to grow along with the outboard of the scaffold with mucosa, submucosa, muscular layer, and the serosa layer order after 7 weeks. Mucosa and muscular layer growth along the scaffold inner wall were seen simultaneously.

**Conclusion:**

3-dimensional electrospinning synthesized 4 : 6 polycaprolactone–silk fibroin nanofiber scaffolds that are feasible for tissue growth and achieve the purpose of ureteral reconstruction in animal experiments. This new form of 3-dimensional electrospinning constructed polycaprolactone–silk fibroin nanofiber scaffold may be considered as a clinical urinary tract tissue reconstruction alternative in the future.

## Main Points

- Proper ratio of the polycaprolactone–silk fibroin (PCL-SF) for scaffold production is achievable for tissue regeneration.
- 3 dimensional (3D)-assisted electrospinning (ES) nanosynthesized PCL-SF scaffolds are biocompatible and durable for ureter tissue regeneration.
- These 3D-assisted ES synthesized PCL-SF scaffolds are feasible for long segment ureteral defect replacement in a preliminary animal study model.

## Introduction

Long segment defect or tissue losses are a difficult issue for recovering or maintaining organ or tissue function in patients with urological diseases, such as severe ureter stricture, small capacity of the bladder, and obstructive urethral injury. Therefore, the design and synthesis of substitutes combined with tissue engineering is an emerging platform for resolving these obstacles. Biomaterials usually are designed to mimic the physical, mechanical, and biological characteristics of the native tissue to facilitate scaffold integration with cell growth and differentiation while simultaneously providing a tolerable degradation rate.^1^

Among all elements of urinary tract, including urinary bladder and urethra, only in the case of ureter, clinical trials involving tissue engineering applications were not been performed so far. Several studies involving using decellularized ureteric grafts, synthetic acellular poly(lactide-co-epsilon-caprolactone) (PLCL), and reinforced collagen–vicryl scaffolds for ureteral defect reconstruction have been reported with equivocal results in animal models due to the possible development of constriction of the scaffold lumen and subsequent hydroureteronephrosis.

In recent years, nanotechnologies have been applied in the production of biomaterial scaffold for providing increased contact area and porous structures that facilitate cellular attachment and growth. Currently, electrospinning (ES) is a mature technique in producing nanoﬁbrous biomaterials.^2,3^ 3-dimensional (3D) bioprinting methodology is a combination of biomaterials and 3D printing skill to replicate units that mimic natural tissues in the human body. Most recently, it has been applied as scaffolds to promote the reconstruction of damaged tissues.^4^ The combination of 3D biomaterials engineering with ES nanotechnology for tissue regeneration is emerging and we presented our preliminary results in synthesizing 3D-ES produced polycaprolactone and silk fibroin (PCL-SF) nanofiber scaffolds for tissue regeneration in the ureteral defects.

## Materials and Methods

Polycaprolactone (PCL, MW 70-90 kDa) and lithium bromide were purchased from Sigma Aldrich (St. Louis, Mo, USA). Formic acid, chloroform, and sodium carbonate were purchased from Merck (Merck KGaA Taiwan Ltd, Taiwan, China). Dialysis membrane was purchased from Thermo Fisher Scientific (Waltham, MA, USA). Bombyx mori cocoons were obtained from a nearby silk farm in New Taipei city. Ethical committee approval was received from Tri-Service General Hospital (TSGH-17-G0-22) and written informed consent was obtained from all participants who participated in this study. 

Bombyx mori cocoons were degummed and processed to form SF according to a previous report.^5^ Briefly, 10 g of cocoons were boiled in 500 mL of 0.02 M Na_2_CO_3_ for 1 hour at 65°C° and then rinsed 4-5 times with ultrapure distilled water. The degummed fibers were kept overnight for drying. Then, the fibers were dissolved in 50 mL of 9 M lithium bromide at 60°C to obtain a viscous solution which was dialyzed for 3 days. The dialyzed solution was lyophilized for 48 hours to obtain SF sponges.

Polycaprolactone and SF were dissolved in 7% (w/v) formic acid, respectively. The solution of PCL and SF were mixed in different ratios of PCL-SF in 100 : 0, 70 : 30, 60 : 40, 50 : 50, 40 : 60, 30 : 70, and 0 : 100, giving a total of 7 different emulsions for electrospinning.^6^ All the emulsions were stirred for 12 hours at 1000 rpm. For electrospinning, a 1 mL capacity of a syringe loaded with emulsion was attached to a 20 G needle tip. 4.0-6.0 L/min flow rate of the emulsion was maintained by a syringe pump, and a voltage of 22-23 kV was maintained between the needle tip and collector with a Taylor cone pattern and cone-jet mode, kept at a distance of 12 cm apart for nanofiber collection. All the experiments were carried out in ambient environments with a humidity of 35%. The electrospun nanofibers were collected and treated using methanol to stabilize the SF structure. Then, the electrospun scaffolds were washed with distilled water for 2 days and air/oven-dried and they were stored in a vacuum.

**Figure 1.**
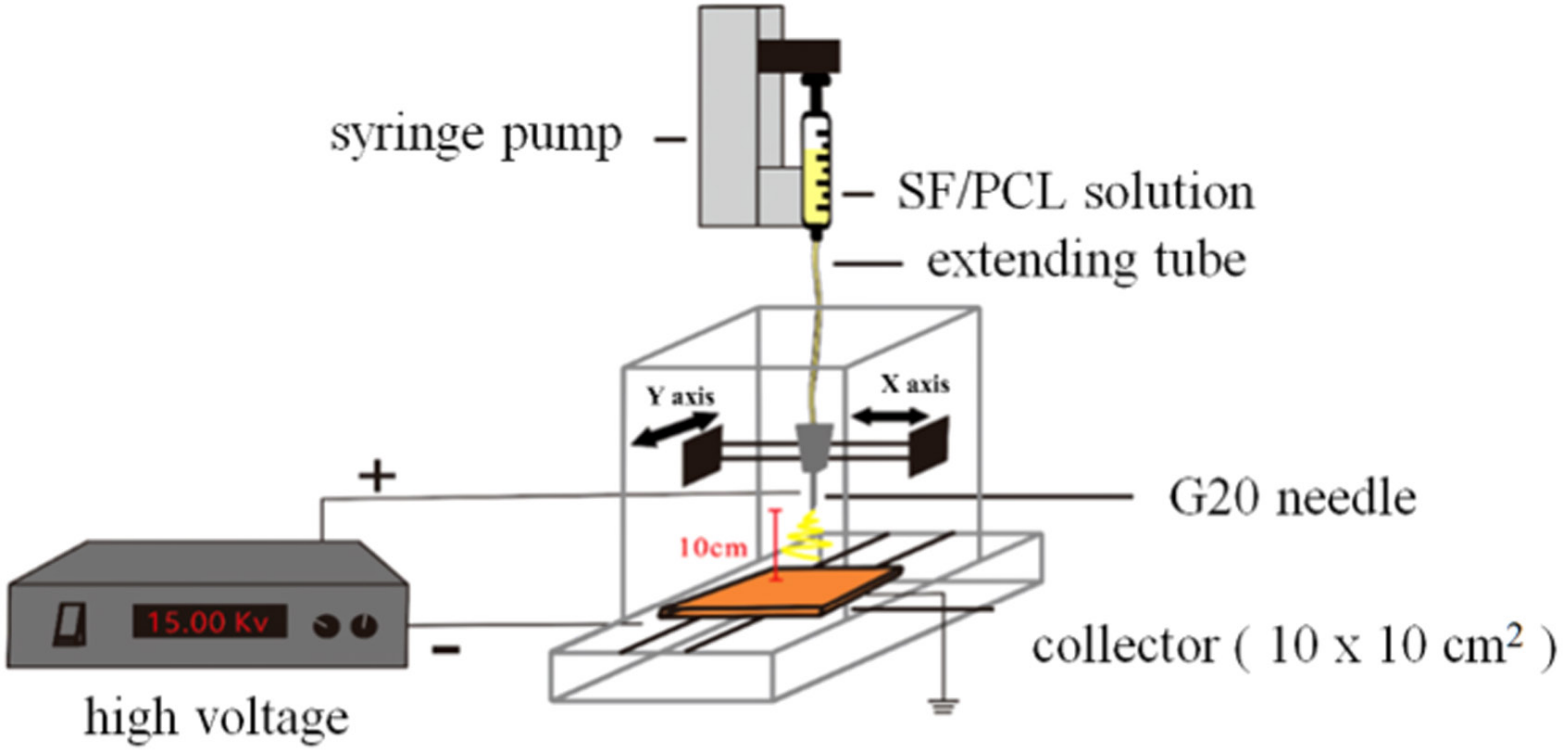
Schematic diagram of the process in established 3-dimensional-assisted emulsion electrospun printing of SF-PCL scaffold. SF PCL, silk fibroin and polycaprolactone.

Polycaprolactone–silk fibroin solution mixture in the syringe was connected to the G20 needle tip in the 3D printer (Prusa I3 3D printer, Prague, Czech Republic) assembled under the adjusted voltages for ES processing which was conducted as 0.4 mL of PCL-SF solution spun at an axial slew rate of 40 mm/sec then followed by 0.1 mL spun at 2 mm/sec.^7-9^ Sprayed nanofibers were formed on the collector with a maximal area surface of 10 × 10 cm^2^ and tip-to-collector distance of 10 cm (Figure 1). Before the implantation study, PCL-SF scaffolds were sterilized using 70% ethanol and ultraviolet and then rinsed in phosphate-buffered saline (PBS) overnight.

The PCL-SF scaffolds were examined under the scanning electron microscope (Carl Zeiss, Oberkochen, Germany) at an accelerating voltage of 15-20 kV. They were mounted on metal stubs and gold-coated in a vacuum for 100 seconds. The fiber diameters and pore size were measured individually.

For in vitro cell culture studies, 3T3 fibroblast cells were cultured in Dulbecco’s modified Eagle medium, and human normal urothelium cells, SV-HUC-1, were cultured in Kaighn's modification of Ham's F-12 medium supplemented with 10% fetal bovine serum (Gibco, Mass, USA) and 1% penicillin–streptomycin under 37^°^C and 5% CO_2_ in an incubator. After sterilization, the 5 mm × 5 mm PCL-SF scaffolds were kept in culture media in 24-well microplate overnight and 5 × 10^5^ 3T3 or SV-HUC-1 cells were seeded in each well supplemented with 500 μL medium and incubated for 1, 2, 3, 5, and 7 days. At each time point, culture scaffolds were taken out and the cells were fixed with 4% paraformaldehyde followed by graded ethanol/propanol treatment and observed under the scanning electron microscope. The cell viability assay on scaffolds was detected using a live/dead assay kit (Life Technologies, Mass, USA).^10^ Briefly, the scaffolds were washed with PBS and incubated with 2 μM calcein-acetomethoxy (AM) and 4 μM ethidium homodimer for 30 minutes. Then, they were washed with PBS and the fluorescence images were examined under an inverted fluorescence microscope (Carl Zeiss, Oberkochen, Germany). 

**Figure 2.**
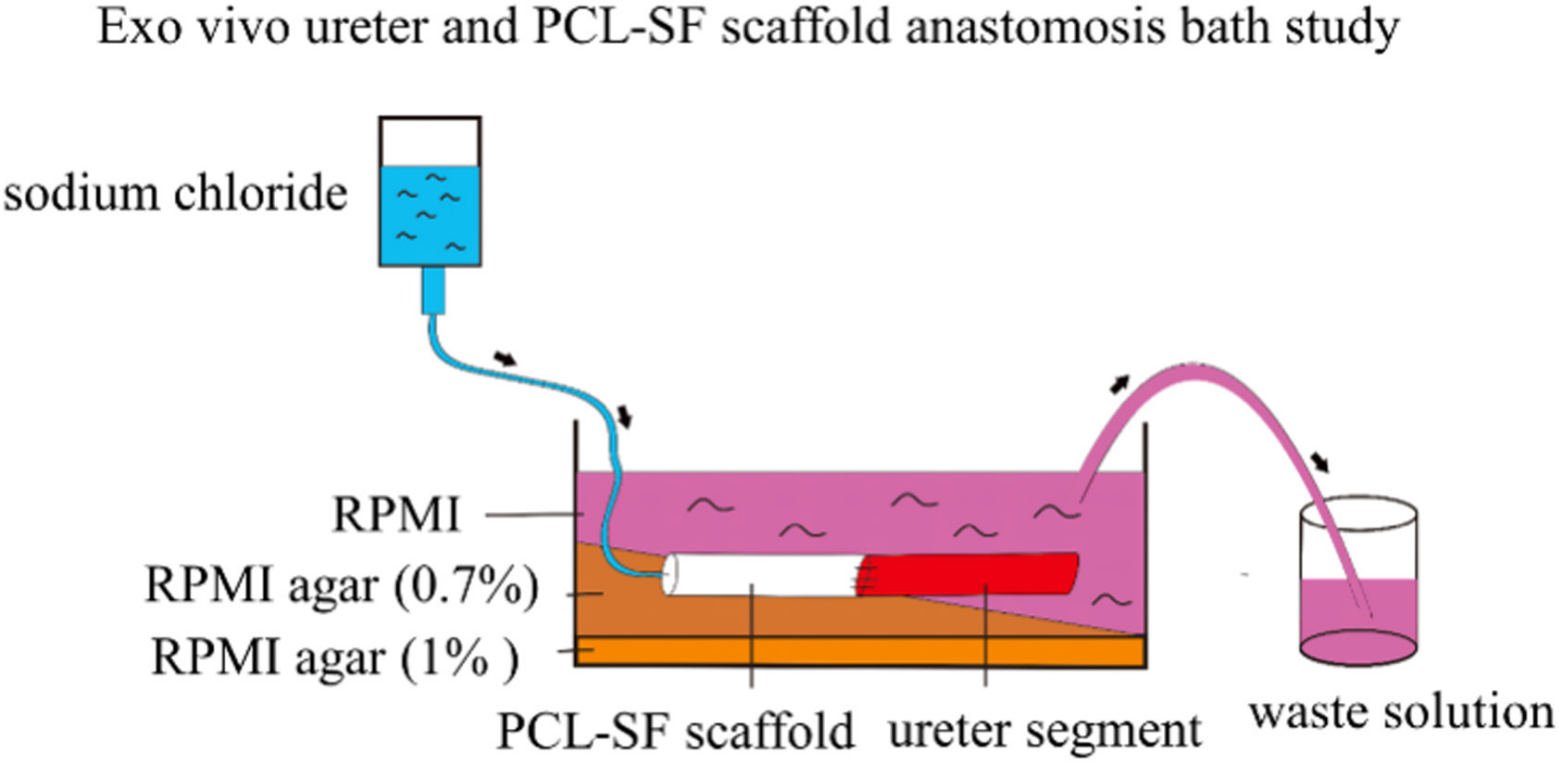
Schematic illustration of long-term ex-vivo agar bath culture of segmental ureter tissue with PCL-SF scaffold anastomosis. PCL-SF, polycaprolactone–silk fibroin.

The ratio 4 : 6 PCL-SF scaffolds were selected as further study materials as it is most feasible for cell growth and is durable in the culture period for at least 7 weeks. A two-layer agar tissue bath was designed with the bottom 1% agar containing Roswell Park Memorial Institute (RPMI)-1640 medium supplemented with 5% fetal bovine serum and 1% penicillin–streptomycin as supporting in 10 cm^2^ petri dish. Healthy ureteral segments, ranging from 1.5 to 2.0 cm, were obtained from patients who received simple nephrectomy with diseased kidneys under their consent and IRB authorization (IRB no.: TSGH-17-G0-22). Rolled PCL-SF scaffolds in tube shape were anastomosed to the ureter segment using 3-O catgut sutures. The tip of the IV set was inserted to the ureteral end and fixed following with constant lumen drip irrigation using 0.95% normal saline solution at a rate of 10-12 mL/h. The scaffold-ureter anastomosis was embedded in the top layer using 0.7% agar and 50 mL of RPMI-FCS culture medium (Figure 2). The medium was changed twice a day. After 14 days, the cultured anastomosis was harvested and examined histologically.

**Figure 3. a,b.**
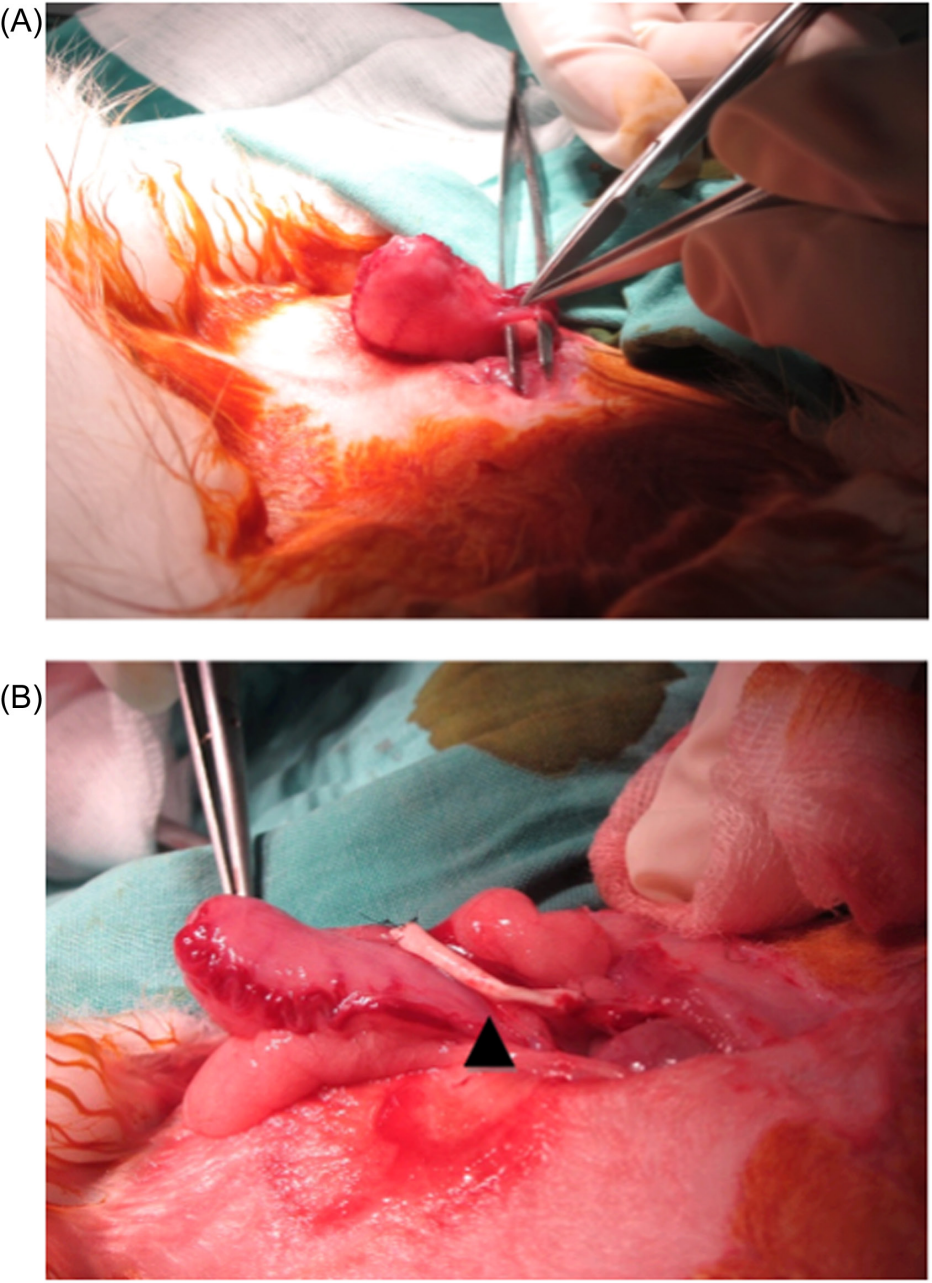
The lower third left ureter was exposed after exploratory laparotomy in New Zealand rabbit (a) and been replaced using PCL: SF (4 : 6) scaffold tube (arrowhead) in one 2 cm resection defect of the ureter (b). PCL-SF, polycaprolactone–silk fibroin.

Three New Zealand male rabbits (7 weeks of age with a weight of 2.0-2.2 kg, National Laboratory Animal Center, Taipei, Taiwan) were subjected to receive ureteral scaffold interposition experiment. General anesthesia was performed by intramuscular injection of Zoetil 50 (tiletamine and zolazepam) (10 mg/kg) and xylazine (5 mg/kg), and a midline low abdominal incision was made after shaving and sterilization. The left ureter was identified and dissected. Then, one 2 cm 4 : 6 PCL-SFP scaffold tube (Length (L) : Width (W) = 20 mm × 1.6 mm, wall thickness of 0.3 mm) was interposed over the low third ureter after the resection of one 2 cm segment native ureter. Bilateral cutting ends of the ureter were anastomosed to scaffold using interrupted 6-O polydioxanone sutures under the microscope (Figure 3). The incised wound was closed with absorbable sutures. Post-operative pain and infection control were controlled with carprofen and antibiotics. At 7 weeks post-ureteral interposition, ureter tissues were harvested for endpoint evaluation. Rabbits were not sacrificed, but only the whole right ureter with scaffold interposition was removed with the ureter end being ligated after general anesthesia and exploration. Removed tissues were fixed in 4% paraformaldehyde and processed for histochemistry study. All animal studies were approved by the National Defense Medical Center Animal Care and Use Committee prior to experiment conduction (no.: IACUC-17-274).

Paraformaldehyde-fixed ureter-scaffold tissues were parafﬁn-embedded, and serial 10 μm sections were cut and then stained with hematoxylin and eosin (H&E) or Masson’s trichrome (MTS).^11^ For MTS stain, tissues were re-fixed in Bouin’s solution after deparaffinization and rehydration. Then, sections were stained using Weigert's iron hematoxylin working solution and Biebrich scarlet-acid fuchsin solution after tap water rinse individually. Finally, tissue sections were treated directly using aniline blue and differentiated in 1% acetic acid. The cellular growth and distribution along the scaffolds were observed and recorded under microscopy.

## Results

**Figure 4. a-h.**
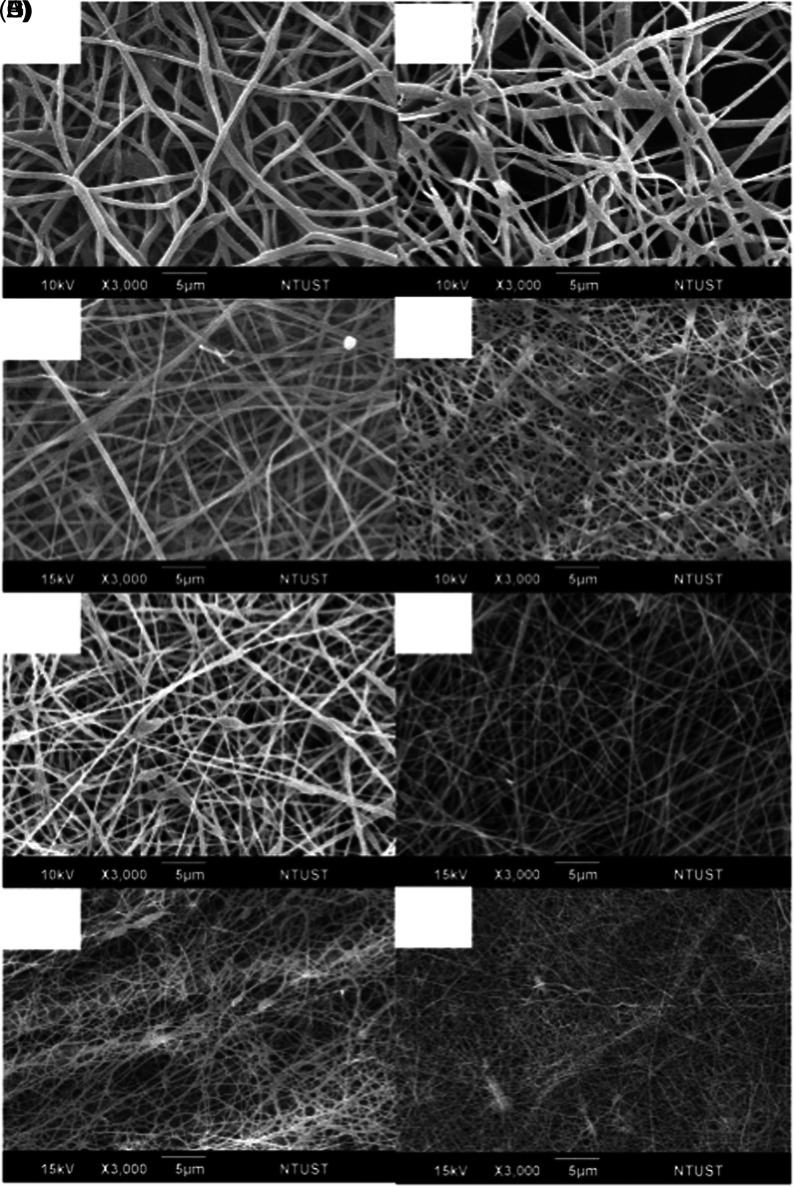
SEM of representing scaffolds in different ratios of SF and PCL (×3000). (a) pure SF, (b) PCL: SF = 3:7, (c) 3D-ES PCL: SF = 4: 6, (d) PC L: SF = 4: 6, (e) PCL: SF = 5: 5, (f) PCL: SF= 6: 4, (g) PCL: SF = 7: 3, (h) pure PCL. Intercalating pores between stereo-nanofibers becoming larger with increased diameter of nanofibers were noted when the component of SF increased as in (a) and (b). 3D-ES PCL: SF (4:6) scaffold has larger pores than ES PCL: SF (4:6) scaffold (c and d). PCL-SF, polycaprolactone–silk fibroin; SEM, scanning electron microscope; 3D-ES, 3-dimensional electrospinning.

Scanning electron microscopy analysis in each PCL-SF group demonstrated that variations in the ratio of PCL and SF led to selective differences in nanofiber diameter and size of pores formation between stereo-nanofibers (Figure 4) (Table 1). When the SF component increased, the diameter of the nanofiber increased with larger pores formation and formation rate. The matrix architecture of PCL-SK (4 : 6) scaffolds between ES and 3D-ES method is similar in deposited silk layers with criss-cross pattern and the latter has a larger diameter of nanofiber (0.70 μm vs. 0.63 μm with 11% decreasing difference in 3D-ES method) and pores (3.26 μm^2^ vs. 2.31 μm^2^ with 41% decreasing difference in 3D-ED methods). The difference may be due to the constant rotational speed that is maintained during 3D printing (Figure 4c and d).

**Figure 5. a-h.**
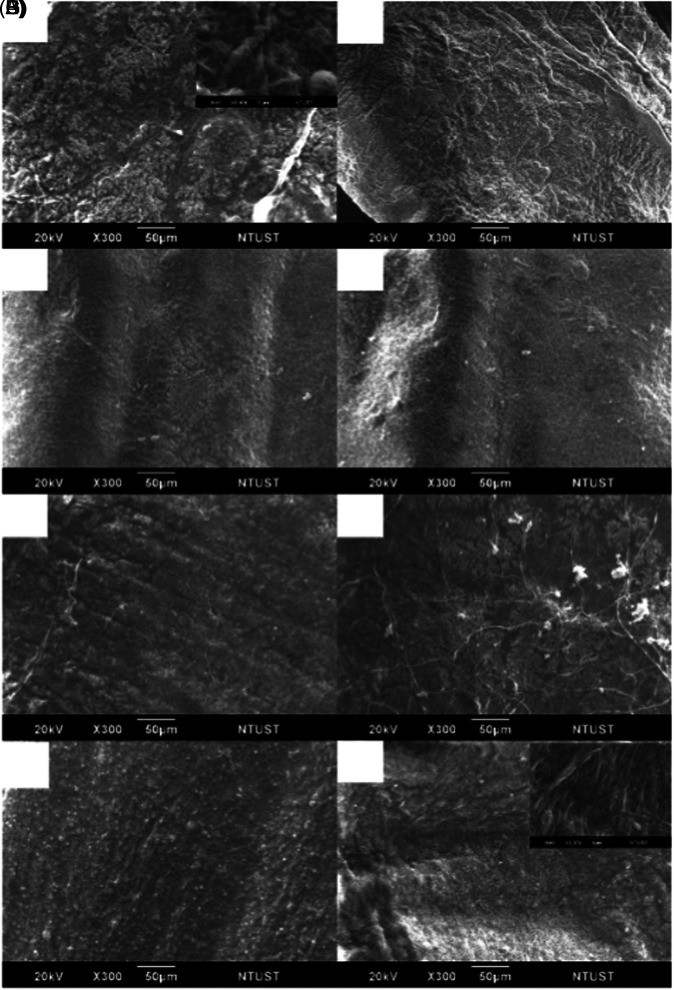
SEM of cultured 3T3 and SV-HUC-1 cells could effectively penetrate inside the nanoﬁbrous scaffolds in 3 days (×300). (a) pure SF, (b) PCL : SF = 3: 7, (c) 3D-ES PCL : SF = 4 : 6, (d) PC L: SF = 4 : 6, (e) PCL: SF = 5 : 5, (f) PCL: SF= 6 : 4, (g) PCL: SF = 7 : 3, (h) pure PCL. PCL-SF, polycaprolactone–silk fibroin; SEM, scanning electron microscope; 3D-ES, 3-dimensional electrospinning.

**Figure 6.**
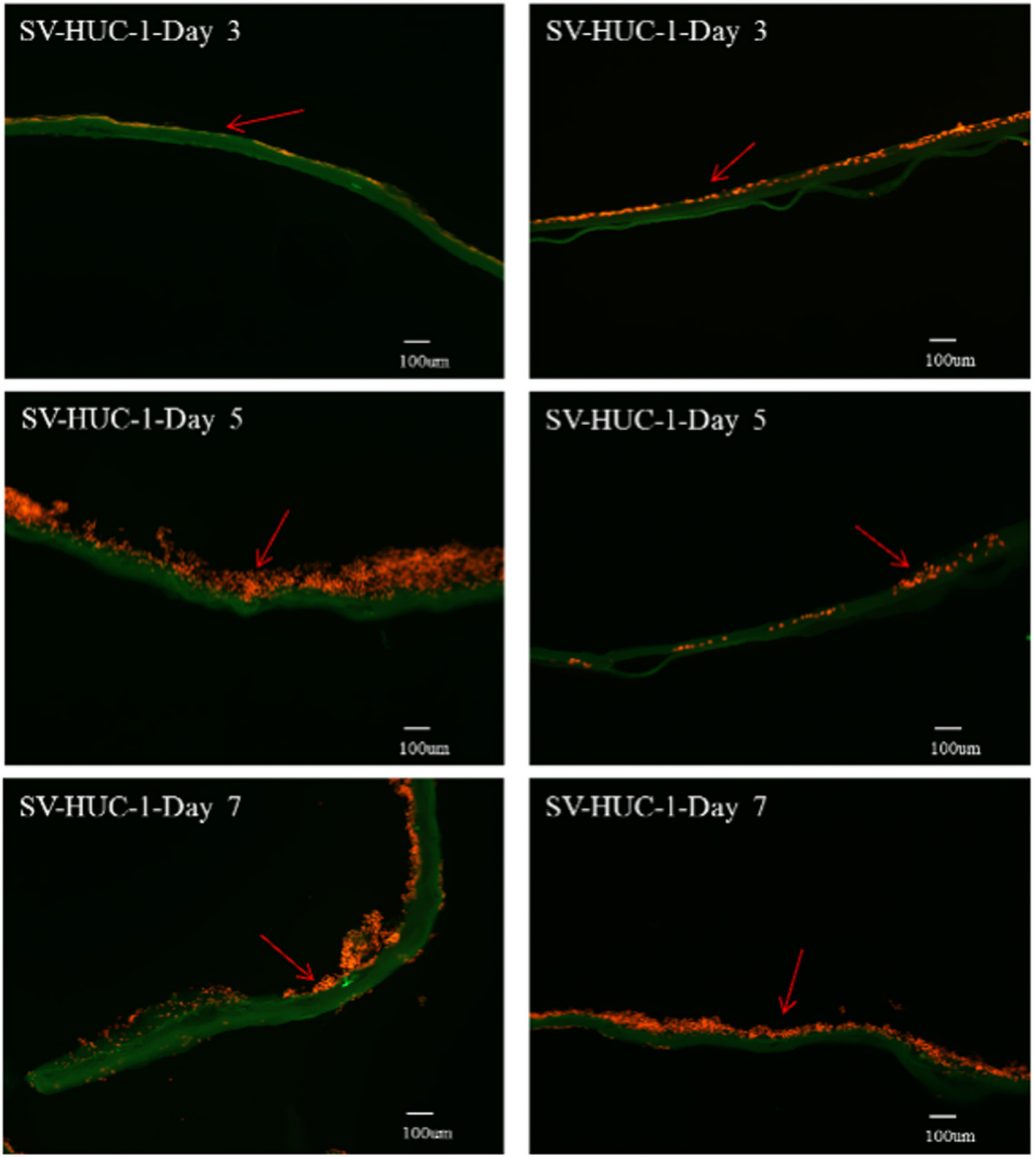
Calcein acetomethoxy and ethidium homodimer stain of SV-HUC-1 uroepithelial cells (15 000 cells) cultured in PCL-SF (4:6) scaffold synthesized by ES (left lane) or 3D-assisted ES methods (right lane) in 3 days, 5 days, and 7 days individually. Cellular growing migration within the scaffolds (arrows indicate red-colored cells) can be seen in 3 days and growth into the counter side can be seen in 5 and 7 days (×40). PCL-SF, polycaprolactone–silk fibroin; 3D-ES, 3-dimensional electrospinning.

The PCL-SF scaffolds presented an excellent water absorption capacity and well elasticity with 60% compressive strain support (data not shown). In vitro culture assays demonstrated that the growing cells were viable and that the cultured 3T3 and SV-HUC-1 cells could electively penetrate inside the nanoﬁbrous scaffolds in 3 days (Figure 5). When the component of SF increased, higher cellular growth was observed in either 3T3 or SV-HUC-1 cells. Under fluorescence stain using calcein-AM and propidium iodide, cultured cells initially can be seen covered on the surface of 3D-ES synthesized PCL : SF (4 : 6) scaffold fluorescently in 3 days, then increased in number within the scaffold in 5 days, and finally distributed over both sides of the scaffold wall in 7 days (Figure 6).

**Figure 7. a-d.**
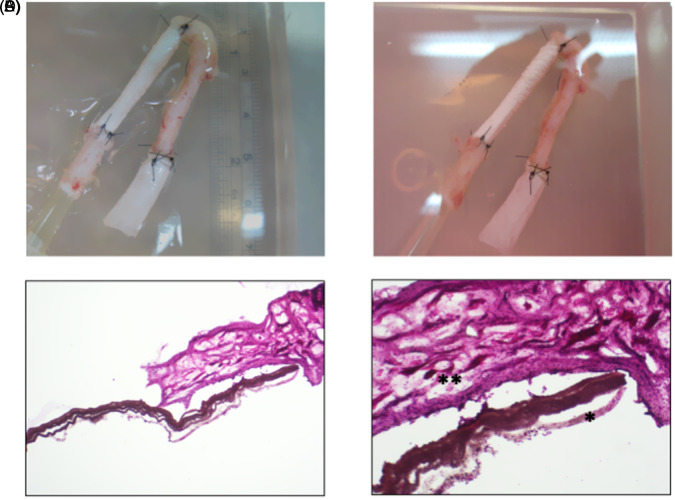
Ex vivo cultured uretero-PCL/SF (4 : 6 3D-ES) scaffold segment in day 1 (a) and in day 14 (b). Histological examination of cultured anastomosis site revealed viable ureteral tissue growing along the scaffold around 1.2 mm in length with epithelium in the inner layer and stromal cells in the outer layer of scaffold (H&E stains, ×40 (c), ×100 (d) (*Mucosa layer, **Sero-muscle layer). PCL-SF, polycaprolactone–silk fibroin; HE, hematoxylin and eosin; 3D-ES, 3-dimensional electrospinning.

As shown in Figure 6, the ex vivo cultured uretero-PCL/SF (4 : 6) scaffold segment was maintained in viable condition for 14 days. Histological examination of cultured anastomosis site revealed healthy and viable ureteral tissue growing along the 3D-ES synthesized PCL-SF (4:6) scaffold around 1.2 mm in length with epithelium in the inner layer and stromal cells in the outer layer of scaffold (Figure 7).

**Figure 8.**
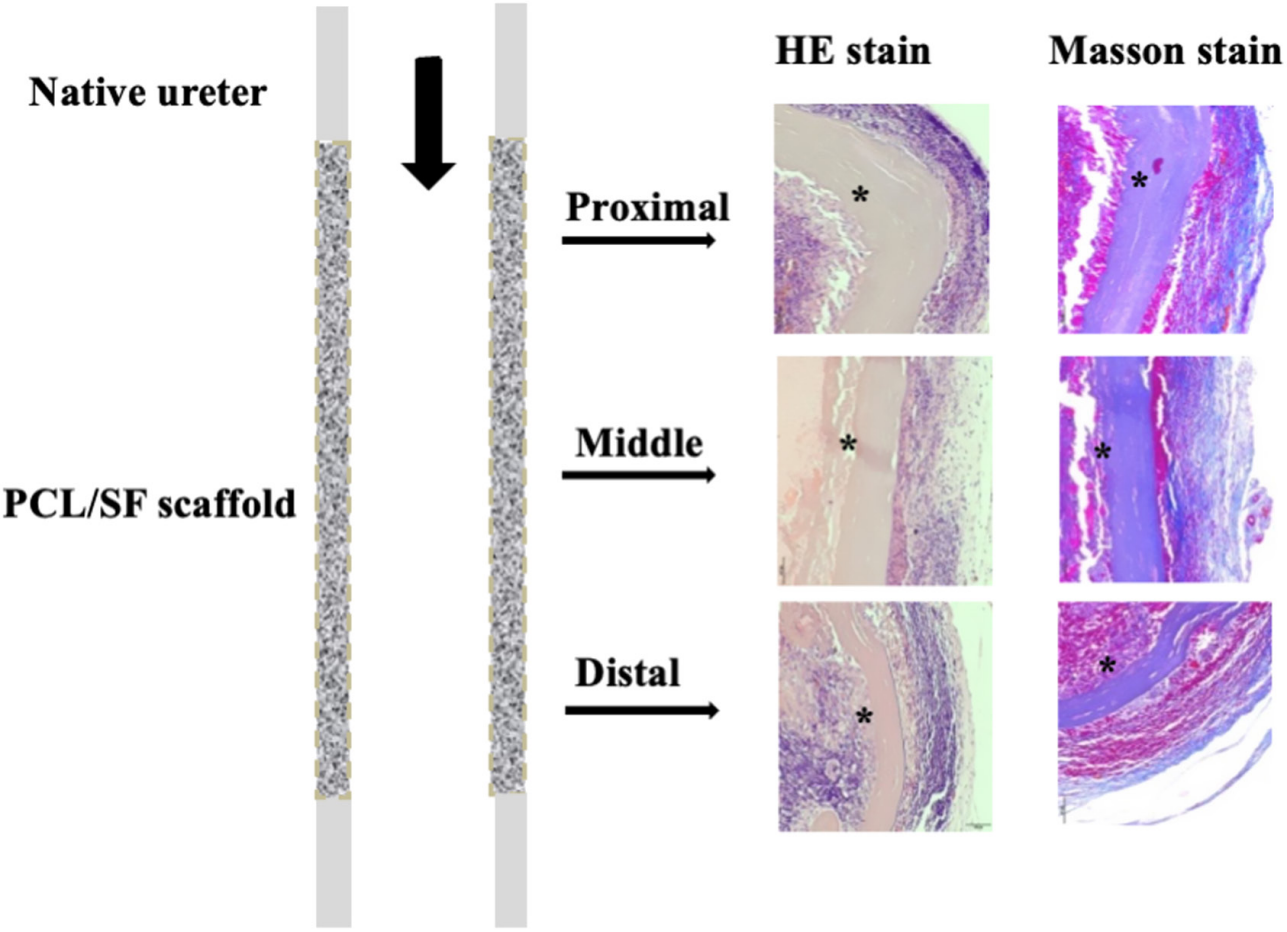
The histological examination of newly developed ureter segment with transplanted PCL-SF (4 : 6) scaffold (2 cm) in the rabbit ureter in 7 weeks (H&E and Masson stain, ×40, transverse view, *Scaffold) (smooth muscle layer is pink in H&E and red in Masson stain). PCL-SF, polycaprolactone–silk fibroin; H&E, hematoxylin and eosin.

The scaffolds formed by 3D-assisted ES using 4 : 6 PCL-SF were successfully implanted in ureteral lesions created in a rabbit model. Seven weeks after implantation, the replaced ureteral defects were ﬁlled with well-organized ureter-like tissue. During exploration, no marked obstructive hydronephrosis or adhesion was seen around the implanted scaffold in rabbits. Gross and microscopic examination of whole scaffold implanted ureter sections revealed that there was a prominent ingrowth of layered ureteral tissue, including mucosa, submucosal, and muscular layers, from the border of the native ureter, that bridged the original defect site. The ingrowth pattern was simultaneous bi-directionally in exterior and interior at 6 and 7 weeks postoperatively (Figure 8). Cellular stratification with inner urothelium, middle muscle, and outer serosa layers alignment was seen fully covered the outer side surface of implanted PCL-SF scaffold. Slower urothelium migration was also observed in the inner surface of scaffold lumen simultaneously at 7 weeks. Hematoxylin and eosin and MTS stains showed that the regenerated ureteral tissues within the scaffolds consisted of a robust smooth muscle layer (pink in H&E; red in MTS) dispersed throughout the periphery of the soft tissue with epithelium coverage (Figure 8). There was no severe inflammatory fibrosis reaction that was observed in the intercalated scaffolds. The majority of the residual PCL-SF scaffold persistently reserved within the original lumen without substantial breakdown and fragmentation occurred at 6 and 7 weeks. The degradation rate of PCL-SF scaffold should be closely related to the exposure area to proteolytic enzymes and polymer hydrolysis. 

## Discussion

The main advantage of combined SF and PCL in using 3D bioprinting-assisted ES is that they can evenly layer by layer distribute emulsified nanofibers by ES at 3D printing and form semipermeable scaffolds. Electrospinning procedure helps nanoparticle formation and 3D printing helps homogenize pore formation in layers.^6,11,12^ Silk fibroin can promote nanoholes formation and PCL function as ureter molding and support during the tissue regeneration process.

This study describes the advances in biomaterials design and production techniques that combined with ES-nano and 3D printing technology, resulting in a ﬁne alignment in micro-and nano-structure, durability, and composition for a better ureter regeneration. There was no prominent degradation change of the PCL-SF scaffold under in vitro long-term culture medium soaking for 8 weeks with only 10% less in dry weight when compared to the original scaffold. The physical tensile power of synthesized PCL-SF nanofibers paralleled increased when the ratio of PCL components increased. Typical spectrum peaks of PCL and SF were observed in the spectra of PCL/SF blends (data not shown). 3-[4,5-dimethylthiazol-2-yl]-2,5 diphenyl tetrazolium bromide (MTT) viable cellular stain demonstrates that the incorporation of SF with PCL is beneficial for cell growth and its growth activity is parallel to the percentage of SF and we found that 4 : 6 of PCL-SF scaffold is the best mixture for cellular growth. Furthermore, higher cellular proliferation was observed in 4 : 6 of 3D-ES combined PCL-SF scaffolds than 4 : 6 of ES-only PCL-SF scaffolds due to increased diameter of nanofibers and size of holes (data not shown). 

In our preliminary study using this new form combined ES-3D methodology, in vitro study demonstrated that cultured cells were viable and they could normally migrate into the nanofibrous scaffold. The scaffold was successfully connected with an ex vivo ureter and produced a normal extended growth pattern along the scaffold. In the rabbit animal model, ureteral defects were filled with uniform and well-organized ureter-like tissue along the PCL-SF scaffold 7 weeks after implantation. In the study, histological assessments revealed substantial differences in the ability of the PCL-SF scaffolds (4 : 6) in driving urothelial formation and maturation of layered tissues along with defect sites in addition to supporting.

The biodegradability of synthetic scaffold is closely related to its durability for complete cellular tissue regeneration. Adjusting the most compliant ES nano-forming and 3-D printing parameters during the processing of scaffold is the most important key factor in the assurance of an effective scaffold that can model the ureteral defect and replace the tissue formation under gradual degradation at the same time. Following the ureter implantation using scaffolds, differentiated and layered urothelium with adequate muscle interposition as native ureter is a critical point in well restoration of the ureteral function. We have demonstrated that the 3D-assisted ES produced 4 : 6 PCL-SF scaffold that can last for at least 7 weeks with intact configuration and patent ureteral lumen in this study. The tubular structure and its maintenance in the ureter functioning as a diversion pass for urine passage are important for ureteral regeneration in addition to its peristalsis activity. 

Regarding the urothelium growth and maturation in the ureter, it is very similar between the ureter and bladder. As a barrier during urine passing through the ureter, the normal proliferation of basal cells with 4-6 layers stratification is essential.^13-15^ The exact mechanisms of smooth muscle regeneration in ureter induced by PCL-SF scaffold are still unknown. In animal bladder model, Pablo Gomez et al^11^ have reported that the involved process in muscle regeneration by SF scaffolds is related to epithelial interactions, submucosal fibroblasts transdifferentiated into smooth muscle cells, or migration of adjacent smooth muscle cells from the host bladder wall into the implanted defect. In our rabbit animal study, H&E and MTS analyses showed that the regenerated ureter wall at 7 weeks implantation already consisted of a robust-layered smooth muscle (pink in H&E; red in MTS) with epithelial covering along with the defect sites, and no marked fibrotic remodeling was seen. 

The research focused on ureteral tissue engineering is limited when compared to that concentrated on the urinary bladder or urethra fields. Most early studies using bare scaffolds in ureteral tissue regeneration had demonstrated poor results due to subsequent severe fibrosis and inflammation reaction in the scaffold, which indicated the necessity of further improving biomaterial components, synthesize technique, and even cell or growth factors addition during processing.^16,17^ In recent years, engineering medicine approaches for ureteral reconstruction have majorly been reported in China and have variable component scaffold designs with different results.^18-23^ They have applied adipose tissue-derived stem cells (ADSCs) with scaffolds that possess the ability to differentiate into epithelial cells and smooth muscle cells.^16^ The materials they used included bladder acellular matrices^18,19,23^ and poly-L-lactic acid collagen nanofibrous.^20-22^ The repaired ureteral defect they used ranged from 0.9 cm in mice to 3-4 cm in rabbits and was observed at 2 weeks and 16 weeks intervals. Fu et al^21^ and Xu et al^22^ utilized composited scaffolds synthesized from poly-L-lactic acid combined with collagen and decellularized matrices. These composited scaffolds offer a feasible surface for cellular attachment and growth and possess good mechanical properties for supporting regenerated tissues. In our study, we synthesized a newly 3D-assisted ES-printed PCL-SF scaffold for promoting native tissue cells growth advancement into the 2 cm length defect in rabbits at 7 weeks. Cellular growth with layered differentiation can be seen in the preliminary study. Recently, Liu et al^24^ have reported that stromal cell-derived factor-1 alpha-treated silk fibroin nanofiber can result in regeneration enhancement of mucosa and submucosal smooth muscles and suppress collagen deposition post-operatively at 3 months in the urethral defect. As cellular seeded pretreatment may enhance the growth rate more, tissue differentiation will be our further study aims in the future using the newly designed PCL-SF scaffold. Nevertheless, other research groups from other countries also have made an attempt to regenerate ureters with tissue engineering methods.^25,26^ Matsunuma et al^25^ have reported using decellularized matrix as scaffold combined with cultured uroepithelial cells with bone marrow-derived mononuclear cells for ureter reconstruction. Recently, Gundogdu et al^26^ have reported using bi-layer silk fibroin grafts for tubular urethroplasty in a porcine defect model with short-term good results.^26^

The unknown is the accurate growth rate of native tissues along the scaffold and final tissue differentiation and organization as native ureter tissue are 2 major concerns and limitations in our study. Theoretically, ureter anatomic recovery by supporting and histological recovery by cellular differentiation can be reached by scaffold combination of PCL and SF. We have observed nearly complete regeneration of ureteral tissue along the 2 cm PCL-SF scaffold in the seventh week with integration and intact of the scaffold. A longer duration of implantation is needed in the further study to confirm the persistency of ureteral patency with urine passage and normalization of ureter tissues with degradation of the scaffold. Further study is also needed to assure the maximal ureteral defect that can be replaced using the PCL-SF scaffold longer than 2 cm (20% of total ureter length in rabbits and estimated 5 cm in the human ureter). Ureter neural network with regular peristalsis function for urine passage is important for regeneration in ureteral defect repair, and cellular scaffold potentially should be better than acellular scaffolds in promoting cellular growth and differentiation after implantation. These are limitations in our preliminary study and are worth further study in the future. Meanwhile, it is reasonable to assume that the regeneration of tissue in rabbits is different when compared to humans. Therefore, the results regarding the time needed in reaching regeneration completeness cannot be extrapolated to humans accurately from this study. Also, sufficient results cannot be achieved definitely with a single animal model and in vitro cell culture experiment in the current study.

In conclusion, 3D-assisted ES-synthesized PCL-SF scaffolds can promote successful urothelial maturation and muscle cells growth at the ureteral defect. Future studies will focus on the kinetics of the regeneration process supported by the PCL-SF scaffold in order to better understand both the initial and long-term effects of scaffold on the implant outcomes.

## Figures and Tables

**Table 1.** Comparison of Diameter, Pore Size, and Pore Formation in Different Ratios of PCL-SF^a^ Scaffolds by ES^b^ or 3D-ES Method

Method				ES				3D-ES
**Ratio of** **PCL-SF**	Pure PCL	PCL: SF (7 : 3)	PCL: SF (6 : 4)	PCL: SF (5 : 5)	PCL: SF (4 : 6)	PCL: SF (3 : 7)	Pure SF	PCL: SF (4 : 6)
Mean diameter of nanofiber (μm)	0.39 ± (0.10-2.88)	0.34 ±(0.10-1.39)	0.44 ±(0.10-1.83)	0.58 ±(0.10-2.38)	0.63 ±(0.10-2.55)	0.85 ±(0.10-4.44)	0.81±(0.10-3.23)	0.70±(0.10-2.48)
Mean pore size (μm**2** **)**	0.78 ±(0.03-32.51)	0.76 ±(0.03-49.71)	1.17 ±(0.03-18.45)	1.18 ±(0.03-17.07)	2.31 ±(0.03-46.90)	4.04 ±(0.03-43.31)	5.27 ±(0.03-66.89)	3.26 ±(0.03-33.31)
**Pore formation (%)**	32.9	41.4	46.0	41.2	40.1	39.7	46.8	45.8

^a^SF, silk fibroin;^b^ES, electrospinning.

PCL-SF, polycaprolactone–silk fibroin.
